# PLANNING AHEAD FOR POSTHOSPITALIZATION DISCHARGE NEEDS AMONG OLDER ADULTS

**DOI:** 10.1093/geroni/igad104.2584

**Published:** 2023-12-21

**Authors:** Raven Relerford, Amber Miller-Winder, Allie Schierer, Charles Olvera, Alaine Murawski, Vanessa Ramirez-Zohfeld, Lee A Lindquist

**Affiliations:** Northwestern University, Chicago, Illinois, United States; Northwestern University, Chicago, Illinois, United States; Northwestern University, Chicago, Illinois, United States; Northwestern University, Chicago, Illinois, United States; Northwestern University, Chicago, Illinois, United States; Northwestern University, Chicago, Illinois, United States; Northwestern University, Chicago, Illinois, United States

## Abstract

Frequently hospitalized older adults require assistance at discharge – either with transfer to a skilled nursing facility (SNF) or additional support at home. Our research has demonstrated that older adults can effectively plan ahead for their discharge needs (e.g., selecting SNFs) prior to hospitalization but some chose not to. Very little is known about what motivates older adults to plan. We sought to identify what variables impact older adults’ post-hospitalization planning. We surveyed a cohort of non-hospitalized adults age 65+ who received PlanYourLifespan.org (PYL), a tool to help older adults plan for their post-hospitalization needs, and then completed follow-up surveys at one month and every 6 months thereafter. Surveys inquire about their post-discharge plans if they were to be hospitalized, specifically rehabilitation/caregiver preferences. Multivariate logistic regression models adjusted for baseline hospitalization decision-making, sex, race, self-efficacy, living status, cognitive impairment, and PYL use. 293 subjects were enrolled (mean 73.5 yrs,40.4% non-White;12 mo. retention rate 94.5%). Subjects were more likely to have plans if they had increased chronic conditions (OR 1.23;p>0.05,1.02-1.47), increased medications (OR 1.15;p< 0.01,1.07-1.25), power of attorney (OR 1.84;p< 0.05,1.01-3.38), and used PYL (OR 2.61;p< 0.01,1.45-4.72). Subjects were more likely to have plans if they had higher medical complexity (OR 1.13 [p< 0.05, 1.03-1.24]), limited health literacy (OR 3.13 [p< 0.05, 1.25-7.81]), and used the PYL website (OR 1.72 [P< 0.05,1.00-2.93]) when asked about planning for future post-hospitalization requiring help in the home. Higher medical complexity, limited health literacy, and use of the planning website were associated with increased hospitalization discharge planning.

